# Intelligent Labeling of Tumor Lesions Based on Positron Emission Tomography/Computed Tomography

**DOI:** 10.3390/s22145171

**Published:** 2022-07-10

**Authors:** Shiping Ye, Chaoxiang Chen, Zhican Bai, Jinming Wang, Xiaoxaio Yao, Olga Nedzvedz

**Affiliations:** 1 School of Information Science and Technology, Zhejiang Shuren University, Hangzhou 310015, China; ysp@zjsru.edu.cn (S.Y.); 600352@zjsru.edu.cn (Z.B.); wjm7878@zjsru.edu.cn (J.W.); 2 International Science and Technology Cooperation Base of Zhejiang Province: Remote Sensing Image Processing and Application, Hangzhou 310015, China; yxx1273412851@163.com; 3 Shulan International Medical School, Zhejiang Shuren University, Hangzhou 310015, China; olga_nedzved@tut.by; 4 Faculty of Biology, Belarusian State University, 220030 Minsk, Belarus

**Keywords:** PET/CT, tumor lesion, registration, fusion, intelligent labeling

## Abstract

Positron emission tomography/computed tomography (PET/CT) plays a vital role in diagnosing tumors. However, PET/CT imaging relies primarily on manual interpretation and labeling by medical professionals. An enormous workload will affect the training samples’ construction for deep learning. The labeling of tumor lesions in PET/CT images involves the intersection of computer graphics and medicine, such as registration, a fusion of medical images, and labeling of lesions. This paper extends the linear interpolation, enhances it in a specific area of the PET image, and uses the outer frame scaling of the PET/CT image and the least-squares residual affine method. The PET and CT images are subjected to wavelet transformation and then synthesized in proportion to form a PET/CT fusion image. According to the absorption of 18F-FDG (fluoro deoxy glucose) SUV in the PET image, the professionals randomly select a point in the focus area in the fusion image, and the system will automatically select the seed point of the focus area to delineate the tumor focus with the regional growth method. Finally, the focus delineated on the PET and CT fusion images is automatically mapped to CT images in the form of polygons, and rectangular segmentation and labeling are formed. This study took the actual PET/CT of patients with lymphatic cancer as an example. The semiautomatic labeling of the system and the manual labeling of imaging specialists were compared and verified. The recognition rate was 93.35%, and the misjudgment rate was 6.52%.

## 1. Introduction

With the development of medical imaging technology, PET (positron emission tomography)/CT (computerized tomography) images play an increasingly important role in diagnosing malignant tumors and other diseases. Clinical experience shows that the combination of PET and CT increases sensitivity and specificity in tumor imaging. Therefore, compared to other detection methods, PET/CT is one of the most accurate [1].

Artificial intelligence technology has driven the development of medical image processing technology. Currently, medical image processing technology mainly uses deep-learning technology. This deep-learning-based intelligent medical imaging diagnosis system needs to prepare a large amount of training data to form a data set so that the system can accurately diagnose injuries. It requires professional medical personnel to label the images one by one accurately. For the system to automatically diagnose lesions in the patient’s organs’ PET/CT images, thousands of labeled PET/CT images are needed as training data sets. This type of labeling is quite labor intensive. Therefore, performing automatic or semiautomatic marking after PET/CT image fusion is crucial.

In the past decades, there have been some combined PET and CT scanning instruments available, which have achieved good results in auxiliary diagnosis [2,3]. However, the corresponding fusion algorithms are not mature enough, resulting in a poor fusion effect. Relevant evidence shows that the two modalities of PET and CT registration and fusion images have a noticeable misalignment [4]. The maximum deviation of the PET/CT fusion image was (4.25 ± 0.26) mm; when the bed load was 85 kg, it was (5.36 ± 0.26) mm. There are mainly two merging methods based on transformation and spatial domain in terms of merging algorithms. Zhou et al. used a wavelet transform-based fusion algorithm to fuse PET/CT images of pancreatic tumors [5]. This method nicely preserved the contour features of the image, but the algorithm was susceptible to layer selection of wavelets. In [6], Mohana et al. used a dual-tree complex wavelet transform (DT-CWT)-based fusion algorithm to fuse PET/CT images of lung tumors, which solves the problems of wavelet translation sensitivity and lack of directionality. Yuanjun et al. [7] used multiwavelets to fuse PET/CT images of lung tumors. The multiwavelet base has a more complex structure and has higher image adaptability than the DT-CWT base. A cross-filter fusion combined geodesic distance concept was proposed in [8] to improve the topology of the fused images. Compared to single transformation domains, mixed transformation domains and spatial domains such as DWT + PCA, SVM + Shearlet, DWT + ICA, Contourlet +PCA, and DWT + Shearlet are relatively effective [9].

Furthermore, Lian et al. [10] proposed a co-clustering algorithm simultaneously to segment 3D tumors in PET-CT images. In [10], the theory of belief functions was adopted in the proposed method to model, fuse, and reason with uncertain and imprecise knowledge from noisy and blurry PET-CT images. The distance metric used to measure clustering distortions and spatial smoothness was changed during the clustering process to ensure that each modality’s segmentation was accurate. Kumar et al. [11] used a new supervised convolutional neural network (CNN) to improve the complementary information fusion performance in multimodal PET-CT.

Image segmentation is the basis for the operation of image labeling. Most clinical practice PET/CT lung image segmentation methods use simple threshold methods, such as the fixed threshold method [12], adaptive threshold method [13,14], etc. The principle of the threshold method is simple, but threshold selection is usually shaped by subjective experience. If the threshold selection is not precise, the accuracy of outlining the target area is significantly reduced. Therefore, the threshold method is not an accurate and robust tumor region delineation algorithm. In addition, there are segmentation algorithms based on region growth [7,15]. This method requires specific marker points (also known as seed points) to be provided in advance to help the algorithm distinguish the foreground (target region, ROI) and background. Automatic image segmentation has poor adaptability, but the effect of its segmentation algorithm is better than the threshold method. Therefore, many aspects of this algorithm still need to be studied further.

There are mainly manual, semimanual, and automatic labeling methods for CT image labeling. Manual labeling consists of visually framing the image to form the separation of the image area. Semimanual and automatic labeling start from the image features and process texture, color, shape, semantics, and other image information before annotating [16,17]. Densely connected deep auto-encoder and density-spectral clustering [18], and a DLBP (deep local binary pattern)-based computerized framework [19] have been proposed to reduce the huge amount of workload that medical experts have to perform in manual data annotation.

Labeling is an important step toward deriving quantitative biomarkers for accurate clinical diagnosis and computer-aided decision support systems. As a result, automatic labeling methods such as convolutional three-dimensional deep supervised networks (3D-DSN) [20,21] and fully convolutional networks (FCN) [22] have been proposed, which have a faster convergence speed and higher recognition ability and achieve better results when labeling CT images automatically. For large-scale medical trials and quantitative image analyses, a method to automatically segment liver and lesions in CT and MRI abdomen images using cascaded fully convolutional neural networks (CFCNs) has been proposed in [23]. Overall, although these automatic methods can greatly reduce the cost of manual intervention, the research on the labeling of lesions in an organ is still rare, and the accuracy of annotation still needs to be greatly improved compared with manual annotation.

In this study, the PET image is enlarged using linear pixel interpolation and enhanced-segmented grayscale transformation to match the size and brightness of the CT image. The least-squares residual affine algorithm is merged by Sym8 wavelet transform to preserve the precise registration of PET and CT images. According to the area with strong absorption of the imaging agent 18F-FDG and the characteristic of the images, the professionals can randomly select a point in the fusion image of PET and CT. The system automatically selects the seed point of the focus area for regional growth, and the lesion is automatically matched and marked on the CT image as a polygon. In this study, the PET/CT of Siemens Biograph 64 PET/CT 52-ring lymphoma cancer patients was taken as an example; the semiautomatic labeling of the system and the manual labeling by imaging specialists were compared and verified. The recognition rate was 93.35% and the misjudgment rate was 6.52%, which indicates good performance.

The paper presents the novel algorithms of PET contrast enhancement, the fusion of PET/CT, and the labeling of the cancer lesions in PET/CT. Based on their knowledge and experience, combined with image features provided by the system, professionals can randomly select one point in the tumor lesion area. Then, our system can automatically segment and mark the lesions. Our method has many benefits. By incorporating the knowledge and experience of professionals, our semiautomatic method is able to achieve superior accuracy when compared to so-called automatic labeling methods. In contrast to fully manual methods, our method can also save a significant amount of effort while maintaining recognition accuracy. It provides an effective way to make training samples for deep learning.

## 2. Materials and Methods

### 2.1. PET/CT Image Labeling Bases and Technical Route

PET uses positron-emitting nuclides to label some physiologically necessary compounds or metabolic substrates, such as glucose, fatty acids, amino acids, receptor ligands, and water. After PET tracer is introduced into the body, a positron scanning machine is used to obtain chemical images of the body. It can show the metabolic activity of organs or tissues and the functional distribution of receptors, called “living biochemical imaging”.

CT scans a layer of a certain thickness of a particular part of the human body with a beam of X-rays. The detector receives the X-rays that pass through the layer, converts them into visible light, and converts them from photoelectricity to electrical signals. The digital converter (digital/analog converter) converts them into numbers; each number in the digital matrix is converted into small grayscale squares ranging from black to white pixels and arranged into an array to form a CT image. Therefore, the CT is a reconstructed image, and different mathematical methods can calculate the X-ray absorption coefficient of each voxel.

^18^F-FDG (fluoro deoxy glucose) refers to fluoro deoxy glucose, and its full chemical name is 2-fluoro-2-deoxy-D-glucose. Because ^18^F-FDG can accurately reflect the glucose metabolism level of organs/tissues in the body, it has become the main imaging agent for PET-CT imaging. Because of the vigorous metabolism of malignant tumor cells, the demand for glucose is increased. After intravenous injection of the glucose analog-^18^F-FDG, most tumor lesions show high uptake of ^18^F-FDG. Standard uptake value (SUV) is a semiquantitative index commonly used to diagnose tumors by PET. The specific value of SUV is the radioactive concentration (kBq/mL)/injection dose (MBq)/body weight (kg) of the lesion. In clinical practice, the SUV value is often used to distinguish malignant tumors from benign lesions, and indicates the degree of tumor malignancy. Many types of literature reports, such as the clinical experience described in [24], proposed that if SUV > 2.5, then it should be considered a malignant tumor, and if SUV is between 2.0 and 2.5, it is considered as a critical range, but if SUV < 2.0, then it can be considered a benign lesion.

In clinical diagnosis, it is often necessary to combine CT images of different modalities with PET images so that one image can simultaneously show the pathological structure (CT image) and the functional metabolism (PET image) of the lesion, which helps medical practitioners learn more about the injury and diagnose the condition. The workload of this combination is enormous; for example, there are 500 PET and CT images of a patient and 1000 DICOM (Digital Imaging and Communications in Medicine) images combined, which require manual matching, registration, and observation. The workload of this effort is insurmountable.

**Figure 1:**
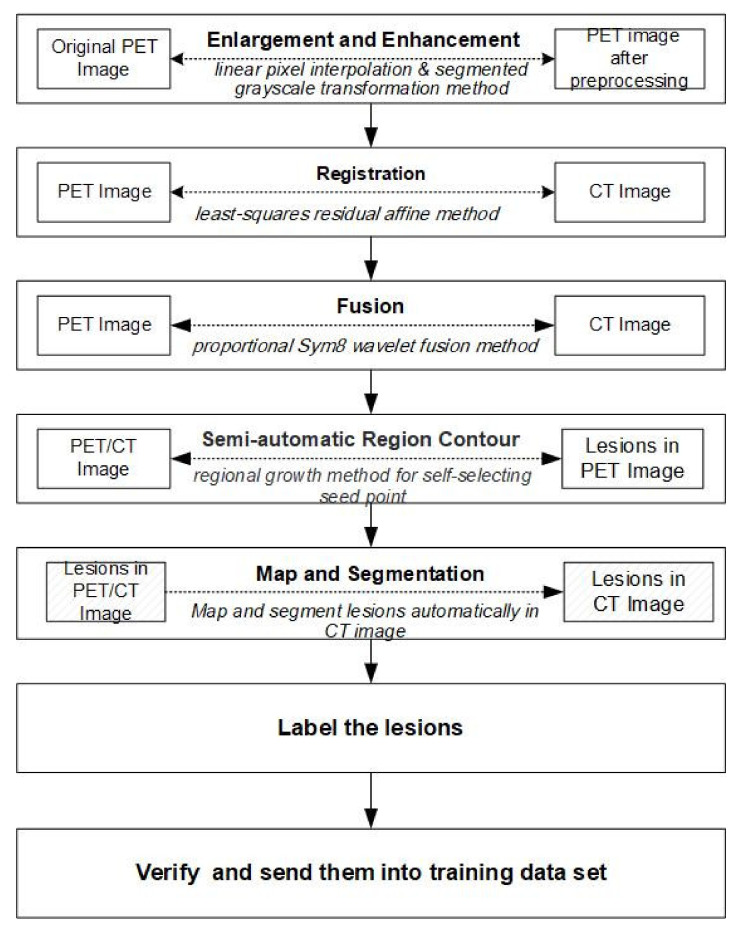
Flowchart of the technical framework.

PET/CT image labeling should undergo PET image enlargement and enhancement, PET/CT image registration and fusion, lesion judgment, and CT labeling. The technical route followed in this article is shown in Figure 1.

### 2.2. Magnification and Enhancement of PET Images

Because of the PET, the image is based on the absorption value of the imaging agent by the body’s tissues and organs; the brightness and clarity of the image are much lower than those of the CT image. Therefore, the PET image must be interpolated, enlarged, and enhanced. PET images have various specifications, such as 128 × 128 and 168 × 168 pixels, while CT images generally have 512 × 512 pixels. The PET image must be enlarged and interpolated to merge with the CT image. Thus, linear interpolation is used to achieve PET magnification of the image size and results in a 512 × 512 image.

The PET images generally show more brightness and less contrast, and the signal intensity is much weaker than the CT images. Nonlinear enhancement of the PET images is required to fuse and display the dots on the fused PET/CT images. Considering that the absorption area of PET is black, that is, a low gray value, to make the gray value more distinguishable and strengthen the detailed features of the low gray area, this study adopts the transformation method of segmentation gray.

Note that the input image is *F*(x,y) and the output image is *G*(x,y). The transformation formula is:

(1) $$G\left(x,y\right)=\left\{\begin{array}{c} 0\;\;\;\;\;\;\;\;\;\;\;\;\;F\left(x,y\right)<PET_{min}\\ \left(\frac{255}{255-PET_{min}}\right)*\left(F\left(x,y\right)-PET_{min}\right)\;F\left(x,y\right)\geq PET_{min} \end{array}\right.$$

### 2.3. Registration of PET/CT Images Base on Least-Squares Residual Affine Method

Because of the different imaging mechanisms of the different PET/CT modalities, the spatial positions cannot be entirely consistent. Therefore, the two images need to be registered. PET/CT image registration is used to match the spatial geometry of CT and PET images. Even if the PET/CT integrated machine is now commonly used, it scans the patient twice: the CT scanning and the PET scanning. Additionally, scanning requires 10–15 min or even longer, so there is a small movement during the scanning because of breathing or heart beating, which results in a slight difference in the image position between the images. Nevertheless, current PET/CT all-in-one machines of all brands are registered in terms of both hardware and software for reconstructing images. At the same time, we cannot use the registration data outside the system. Therefore, to match the spatial geometry of the CT and PET images, registration of the PET/CT images is needed. To register the PET/CT, spatial transformation-based [25] or curve transformation-based [26] image registration techniques have been proposed. To obtain accurate results for the following, the affine least-squares residual method is used to register PET/CT images. The algorithm follows:

Let $A\in (x_{1},y_{1}),(x_{2},y_{2}),\ldots ,(x_{n},y_{n})(n\geq 2)$ be the characteristic points of the image l1, and the characteristic points of the image l2 is $B\in (x_{1}^{\prime },y_{1}^{\prime }),(x_{2}^{\prime },y_{2}^{\prime }),\ldots ,(x_{n}^{\prime },y_{n}^{\prime })$; B is translated from A as follows:

Let the average coordinate adjustment be (*h, k*), then

(2) $$\left\{\begin{array}{l} x_{i}^{\prime }=ax_{i}+by_{i}+h\\ y_{i}^{\prime }=cx_{i}+dy_{i}+k \end{array}\right.\;,\;(i=1,2,\ldots ,n)$$

Therefore, the translation residue at each point can be defined as:

(3) $$\left\{\begin{array}{l} r_{xi}=ax_{i}+by_{i}+h-x_{i}^{\prime }\\ r_{yi}=cx_{i}+dy_{i}+k-y_{i}^{\prime } \end{array}\right.\;,\;(i=1,2,\ldots ,n)$$

Let *C*(*a*, *b*, *c*, *d*, *h*, *k*) be the sum of the squares of the translation residuals of the characteristic point, then

(4) $$C(a,b,c,d,h,k)=\sum_{i=1}^{n}{{[r}_{xi}(a,b,h)]}^{2}+\sum_{i=1}^{n}{[r_{yi}(c,d,k)]}^{2}$$

Let (a,b,c,d,h,k) = $\left(a_{0},b_{0},c_{0},d_{0},h_{0},k_{0}\right)$ and minimize C(a,b,c,d,h,k), then

$$\left\{\begin{array}{l} \frac{dC}{da}{|}_{\left(a_{0},b_{0},h_{0}\right)}=0\\ \frac{dC}{db}{|}_{\left(a_{0},b_{0},h_{0}\right)}=0\\ \frac{dC}{dh}{|}_{\left(a_{0},b_{0},h_{0}\right)}=0\\ \frac{dC}{dc}{|}_{\left(c_{0},d_{0},k_{0}\right)}=0\\ \frac{dC}{dd}{|}_{\left(c_{0},d_{0},k_{0}\right)}=0\\ \frac{dC}{dk}{|}_{\left(c_{0},d_{0},k_{0}\right)}=0 \end{array}\right.$$

Then, the translation can be defined as AX=b. The matrix *A*, the vector *X* and *b* can be defined as follows:

$$A_{2n\times 6}=\left(\begin{array}{cccccc} x_{1} & y_{1} & 1 & 0 & 0 & 0\\ 0 & 0 & 0 & x_{1} & y_{1} & 1\\ x_{2} & y_{2} & 1 & 0 & 0 & 0\\ & & & x_{2} & y_{2} & 1\\ & & \vdots & & & \\ x_{n} & y_{n} & 1 & 0 & 0 & 0\\ 0 & 0 & 0 & x_{n} & y_{n} & 1 \end{array}\right),X={\left(a,b,h,c,d,k\right)}^{T},b={\left(x_{1}^{\prime },y_{1}^{\prime },x_{2}^{\prime },y_{2}^{\prime }\ldots ,x_{n}^{\prime },y_{n}^{\prime }\right)}^{T}$$

Therefore, $A^{T}AX=A^{T}b$

(5) $$X={\left(A^{T}A\right)}^{-1}A^{T}b$$

Then we can obtain

(6) $$\{\begin{array}{c} a_{0}=\frac{\left(\sum_{i=1}^{n}x_{i}x_{i}^{\prime }-n\bar{x}{\bar{x}}^{\prime })(\sum_{i=1}^{n}y_{i}^{2}-n{\bar{y}}^{2})-(\sum_{i=1}^{n}x_{i}^{\prime }y_{i}-n{\bar{x}}^{\prime }\bar{x})(\sum_{i=1}^{n}x_{i}y_{i}-n\bar{x}\bar{y}\right)}{(\sum_{i=1}^{n}x_{i}^{2}-n{\bar{x}}^{2})(\sum_{i=1}^{n}y_{i}^{2}-n{\bar{y}}^{2})-{\left(\sum_{i=1}^{n}x_{i}y_{i}-n\bar{\mathrm{xy}}\right)}^{2}}\\ b_{0}=\frac{\left(\sum_{i=1}^{n}x_{i}^{\prime }y_{i}-n{\bar{x}}^{\prime }\bar{y})(\sum_{i=1}^{n}x_{i}^{2}-n{\bar{x}}^{2})-({\sum_{i=1}^{n}x_{i}x_{i}^{\prime }}_{i}-n{\bar{x}}^{\prime }\bar{x})(\sum_{i=1}^{n}x_{i}y_{i}-n\bar{x}\bar{y}\right)}{(\sum_{i=1}^{n}x_{i}^{2}-n{\bar{x}}^{2})(\sum_{i=1}^{n}y_{i}^{2}-n{\bar{y}}^{2})-{\left(\sum_{i=1}^{n}x_{i}y_{i}-n\bar{x}\bar{y}\right)}^{2}}\\ c_{0}=\frac{\left(\sum_{i=1}^{n}y_{i}y_{i}^{\prime }-n\bar{y}{\bar{y}}^{\prime })(\sum_{i=1}^{n}y_{i}^{2}-n{\bar{y}}^{2})-(\sum_{i=1}^{n}x_{i}y_{i}^{\prime }-n{\bar{\mathrm{xy}}}^{\prime })(\sum_{i=1}^{n}x_{i}y_{i}-n\bar{x}\bar{y}\right)}{(\sum_{i=1}^{n}x_{i}^{2}-n{\bar{x}}^{2})(\sum_{i=1}^{n}y_{i}^{2}-n{\bar{y}}^{2})-{\left(\sum_{i=1}^{n}x_{i}y_{i}-n\bar{x}\bar{y}\right)}^{2}}\\ d_{0}=\frac{\left(\sum_{i=1}^{n}y_{i}y_{i}^{\prime }-n\bar{y}{\bar{y}}^{\prime })(\sum_{i=1}^{n}x_{i}^{2}-n{\bar{x}}^{2})-(\sum_{i=1}^{n}x_{i}y_{i}^{\prime }-n{\bar{\mathrm{xy}}}^{\prime })(\sum_{i=1}^{n}x_{i}y_{i}-n\bar{x}\bar{y}\right)}{(\sum_{i=1}^{n}x_{i}^{2}-n{\bar{x}}^{2})(\sum_{i=1}^{n}y_{i}^{2}-n{\bar{y}}^{2})-{\left(\sum_{i=1}^{n}x_{i}y_{i}-n\bar{\mathrm{xy}}\right)}^{2}}\\ h_{0}={\bar{x}}^{\prime }-a_{0}\bar{x}-b_{0}\bar{y}\\ k_{0}={\bar{y}}^{\prime }-c_{0}\bar{x}-d_{0}\bar{y} \end{array}$$

where $\bar{x}=\frac{1}{n}\sum_{i=1}^{n}x_{i},{\bar{x}}^{\prime }=\frac{1}{n}\sum_{i=1}^{n}x_{i}^{\prime },\bar{y}=\frac{1}{n}\sum_{i=1}^{n}y_{i},{\bar{y}}^{\prime }=\frac{1}{n}\sum_{i=1}^{n}y_{i}^{\prime }$.

### 2.4. Fusion of PET/CT Images Based on the Symlets Wavelet

PET/CT is not a simple combination of PET and CT; it fully exploits the scanning accuracy and attenuation correction accuracy of the CT subsystem and the metabolic imaging of the PET subsystem for quantitative diagnostic lesions. The fast volume acquisition of multislice-spiral computed tomography enables the PET/CT to perform acquisition and attenuation correction, achieve the purpose of high-precision quantitative analysis, and compensate for the shortcomings of low-resolution PET imaging. Before using PET/CT, we have to combine the two. Image fusion generally adopts the wavelet transformation method.

**Figure 2:**
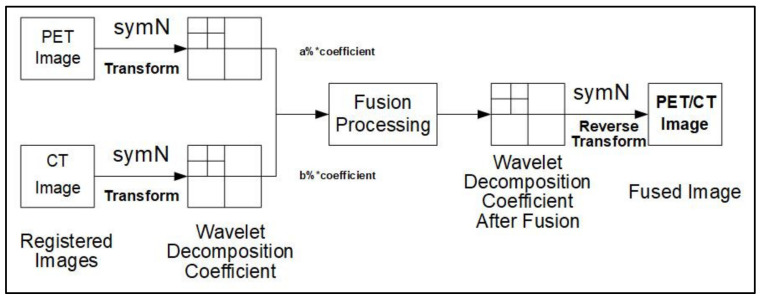
The symN wavelet fusion process for PET/CT images.

This study used the symlets wavelet to merge recorded PET and CT. The symlets wavelet function, proposed by Ingrid Daubechies, is an improved function of DB and an approximately symmetric wavelet function. The symlets wavelet system is usually denoted as symN (N = 2, 3, …, 8). The support range of the symN wavelet is (2N − 1), the runaway moment is N, and it also has reasonable regularity. Because the wavelet has better symmetry, the phase distortion during signal analysis and reconstruction can be reduced to a certain extent. The fusion process of symN wavelets from PET and CT images is shown in Figure 2.

The wavelet transform is to generate the corresponding scale and shift function through a wavelet basis, which is defined as:

(7) $$\Psi (s,\;x)\left(t\right)=\frac{1}{\sqrt{s}}\Psi \;\left(\frac{t-x}{s}\right)$$

where *s* and *x* represent the scale and displacement factors, respectively. The wavelet transform of the information *f*(*t*) is defined as follows:

(8) $$W_{\left(s,x\right)}=\int_{R}\Psi_{\left(s,x\right)}\left(t\right)f\left(t\right)dt.$$

PET and CT images are discontinuous signals. Here, *s* and *x* are discrete forms. After performing power-series processing on *s* and uniformly discretizing *x*, the wavelet transform can be defined as:

(9) $$w\left(a_{0}^{i},kb_{0}\right)=\int_{R}\Psi_{\left(a_{0}^{i},kb_{0}\right)}\left(t\right)f\left(t\right)dt,\;i=0,1,2,\ldots ,k$$

Then, the Symlets wavelet can be expressed as:

(10) $${\left|m_{0}\left(\omega \right)\right|}^{2}={\left[COS^{2}\left(\frac{\omega }{2}\right)\right]}^{N}P\left[sin^{2}\left(\frac{\omega }{2}\right)\right],$$

where *P*(*y*) and $m_{0}\left(\omega \right)$ are defined as follows:

(11) $$P\left(y\right)=\overset{N-1}{\underset{K=0}{\sum }}C_{k}^{N-1+k}y^{k},\;m_{0}\left(\omega \right)=\frac{1}{\sqrt{2}}\overset{2N-1}{\underset{k=0}{\sum }}h_{k}\;e^{-jk\omega }$$

Considering $z=e^{i\omega }$ as a function of *W*, so *W* can be decomposed in different ways as:

(12) $$W\left(z\right)=U\left(z\right)\bar{U}\left(1/z\right)$$

**Figure 3:**
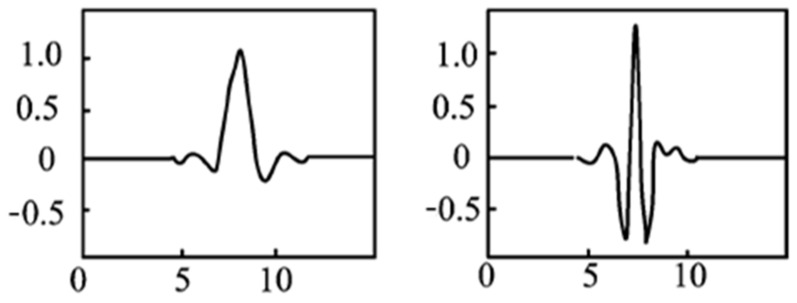
Symlets wavelet scaling function and wavelet function (generated by Matlab).

Their roots are paired; that is, *z* and 1/*z* are both roots. Choosing *U* so that all the module roots are greater than 1, the symlets waveform filter is obtained. The scale function and the wavelet function of the symlets wavelet are shown in Figure 3.

This study uses the eighth-order symlets wavelet function, and the sampling method is a soft threshold. When the decomposition coefficients of PET and CT are synthesized, the ratios of a% and b% are used to obtain the best value in the experiments. After fusion, pseudo color processing was applied to the images to highlight areas of strong ^18^F-FDG uptake (low gray value) in the PET image to aid visual distinction.

### 2.5. Segmentation and Labeling of Lesions in PET/CT Images

After merging the PET/CT image, the practitioner can click on the metabolically active area with the abnormally high SUV value shown on the PET image. The system automatically finds the starting point on the PET image and performs regional growth from the lesion area. The system then maps the PET lesion area boundary to the separated CT and fused PET/CT images, and automatically segments and labels the area on the two types of images.

#### 2.5.1. Regional Growth of PET Lesions Based on Semiautomatic Seed Selection

**Figure 4:**
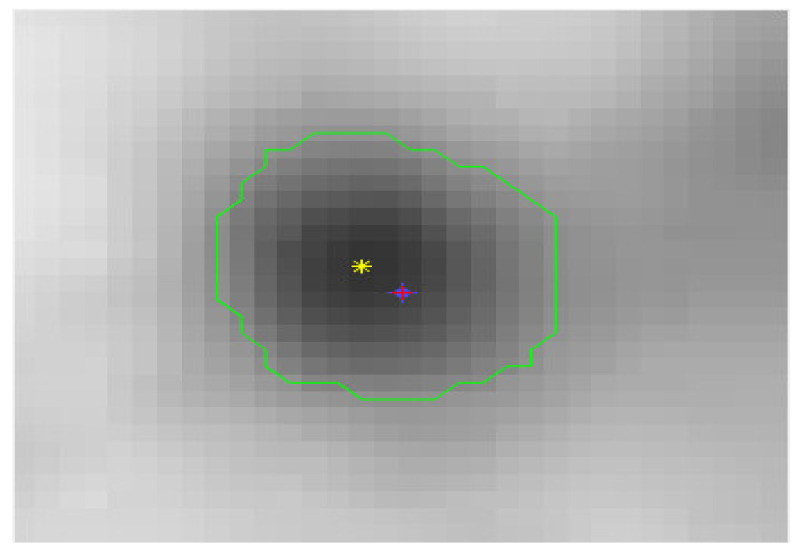
Label point (*x*_0_, *y*_0_) and seed point (*x*_Min_, *y*_Min_). The pink plus is the label point, the yellow star is the seed point, and the green line is the boundary line of the lesion after regional growth.

The system window provides three corresponding PET/CT fusion and CT images. Imaging professionals compare tissues and organs from PET/CT fusion images. In the area of low gray value of PET (strong uptake of ^18^F-FDG), which is regarded as a lesion, the naked eye roughly estimates that when the mouse is clicked, the system finds the point of minimum gray value within a specific range of −8~+8 pixels as the starting point for regional growth, and increases the gray threshold value by 100%, that is, twice the minimum threshold. Eight-field regional growth is then performed to outline the area of the injury automatically. The steps for realizing the growth of the 8-neighborhood region follow:

(1) Click the position coordinate of the PET image and register the pixel coordinate as (x_0_, y_0_). In the range −8~+8 pixels from this point, the minimum gray value is ValueMin, and the position coordinates (x_Min_, y_Min_) of the minimum value are registered. This point is used as the starting point.
(2) Taking (x_Min_, y_Min_) as the center, consider the 8 neighborhood positions (x, y) of (x_Min_, y_Min_), if the value of the coordinate point (x, y) satisfies the growth criterion (gray value < ValueMin + ValueMin* 100%), combine the (x, y) coordinates with the (x_Min_, y_Min_) coordinates (within the same area), i.e., push the (x, y) coordinates onto the stack.
(3) Pop the (x, y) coordinate sequence from the stack, take it as a new growth point, and grow 8 neighborhoods. The coordinates that meet the growth criteria are pushed onto the stack and repeat step 3).
(4) The growth stops when the range of the growth space (x_Min_, y_Min_) is 10 pixels up, down, left, and right; otherwise, return to step 3). After obtaining the lesion area by PET, all the coordinates of the area boundary can be obtained. After registration, PET, CT, and PET/CT coordinates are the same, so PET/CT and CT can divide the lesion area according to the coordinates, as shown in Figure 4.

#### 2.5.2. Delineation of Rectangles and Labeling of Lesions

**Figure 5:**
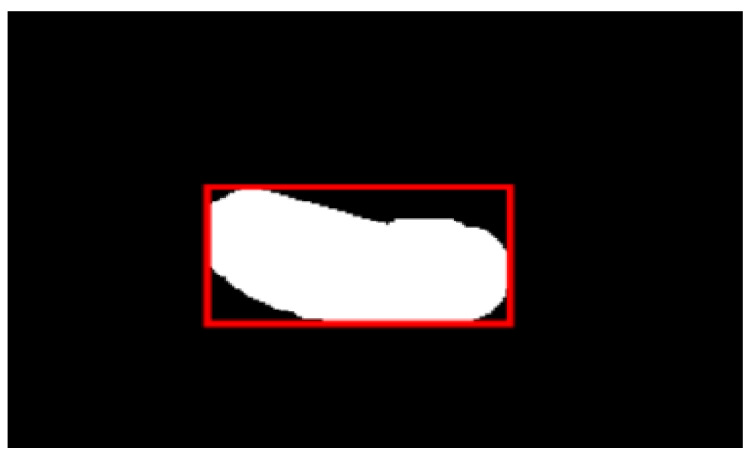
Construction of a rectangular area containing tumor lesions.

The segmented image is an irregular polygon since the deep-learning sample target is a rectangle. In this study, the points where the minimum and maximum values of x and y are first collected, namely (x_min_, y_n_), (x_max_, y_m_), (x_n_, y_min_), (x_m_, y_max_); then (x_min_, y_n_), (x_max_, y_m_) are vertical and (x_n_, y_min_), (x_m_, y_max_) are horizontal, forming a rectangle, which can be formed with (x_min_, y_max_), (x_max_, y_max_), (x_max_, y_min_), (x_min_, y_min_) as the vertices of the rectangle, as shown in Figure 5.

## 3. Results

### 3.1. Experimental Environment

Our experiments and comparisons were implemented in Matlab R2019b on an Inter(R) Xeon(R) Gold 5117 CPU @2.00 GHz server with 256 GB of memory, running Windows Server 2016. In this article, the “Intelligent PET/CT Fusion Labeling System” is developed, which provides users with relatively intelligent labeling of fused PET/CT tumor lesions with only a few interface interactions.

### 3.2. Experimental Process

**Figure 6:**
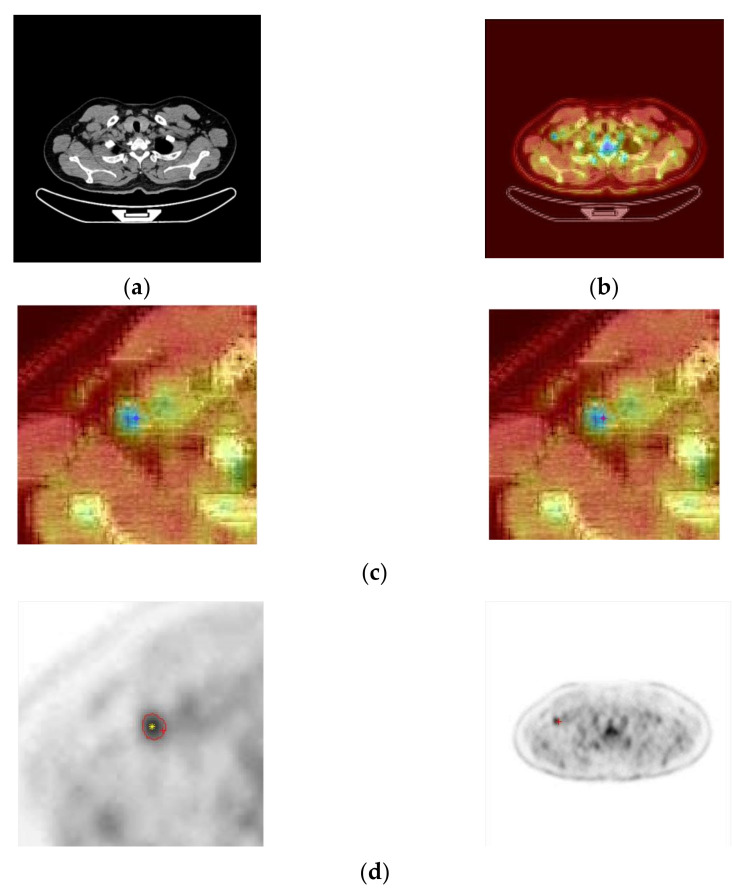
Example of intelligent labeling. (**a**) The original CT image. (**b**) The PET image is fused with the CT image. (**c**) The red “+” symbol on the pseudo color PET and CT fusion image after click. (**d**) The corresponding * symbol appears on the enhanced pet.

(1) Preprocessing: Enter the series of n PET and CT images to be labeled into the system and automatically number them to form PET_1_, PET_2_, PET_3_… PET_n_ and CT_1_, CT_2_, CT_3_… CT_n_.
(2) PET image enlargement and enhancement: The system reads PET_i_ and automatically enlarges it to 512 × 512 pixels through linear interpolation and enhancement by using grayscale segmented transformation.
(3) Registration of PET and CT images: The system moves the corresponding PET_i_ and CT_i_ images to overlay them on the frame, then takes 4–5 points and moves them to PETi by the least-squares residual method until the positions of the two sides are precisely the same.
(4) Fusion test of PET and CT images: Take a pair of PET_i_ and CT_i_, a and b, respectively, take a1 = 30%, b1 = 70%; a2 = 40%, b2 = 60%; a3 = 50%, b3 = 50%; a4 = 60%, b4 = 40%; a5 = 70%, b5 = 30%; after the system auto merges, professionals judge the F(a_1_,b_1_), F(a_2_,b_2_), F(a_3_,b_3_), F(a_4_,b_4_), F(a_5_,b_5_) fusion images, select the fusion image that is most suitable for labeling, and then determine the a and b fusion values, and merge all the PET and CT according to this ratio image pair.
(5) Lesion Labeling Test: Extract the merged image Fi, compare the PETi and CTi displayed in the lower window of the system, and click the tumor lesion area on the PETi (only need to click once in this area; it is not necessary to draw polygons or find the box), as shown in Figure 6.

The system automatically grows regions on the PETi to form a polygonal lesion region. Then, the system automatically maps the polygonal area to the corresponding position of the CTi, segments the lesion, and generates a rectangular box, which is requested to be labeled (Label) in the segmentation map. Of course, if the professionals feel that something is wrong during the process, they can re-operate on the area of the injury.

(1) Repeat step (4) to the end of all pairs of PET and CT images we want to annotate.

### 3.3. Experimental Results

**Figure 7:**
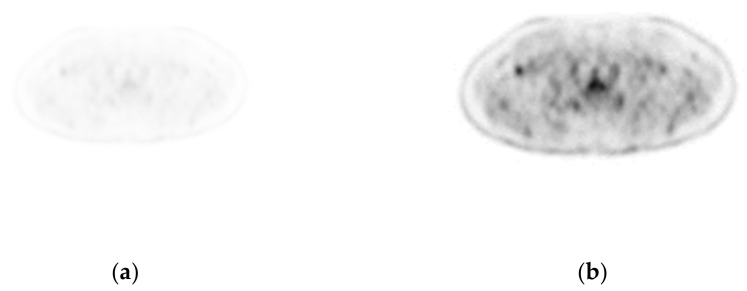
PET image enhancement. (**a**) The original PET image (168 × 168). (**b**) The enhanced PET image (512 × 512).

**Figure 8:**
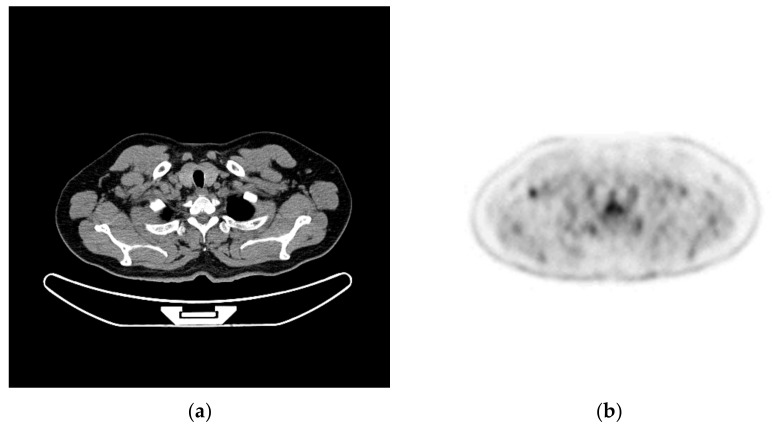
Registration of PET and CT. (**a**) CT image. (**b**) Enhanced PET image.

**Figure 9:**
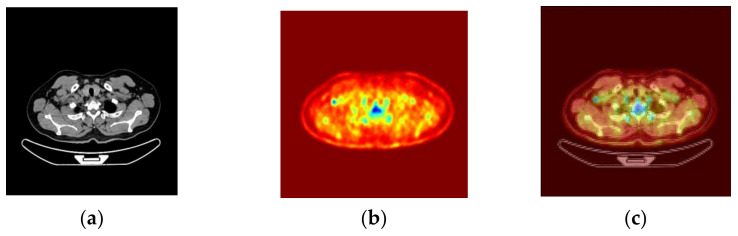
Fusion of PET and CT. (**a**) CT image. (**b**) PET pseudo-color image with 256 colors. (**c**) The PET image is fused with the CT image.

**Figure 10:**
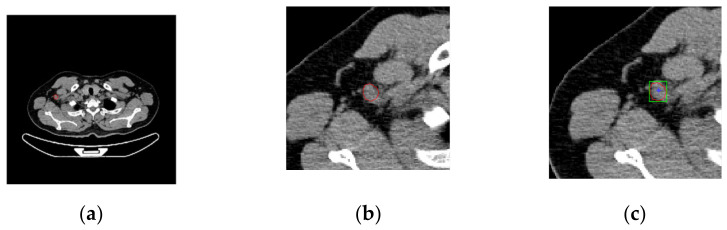
Labeling of lesion segmentation. (**a**) Delineated lesion on the CT image. (**b**) Local enlarged view of delineated focus. (**c**) Divide the lesion with a rectangular box.

(1) Enlargement and enhancement of PET images:
  The enlarged and enhanced effect is shown in Figure 7.
(2) PET and CT registration:
  Taking the CT image as a reference point, after using the gantry registration, it is found that there is still a tiny gap between the CT and the enlarged and enhanced PET image. A good record is obtained after implementing the least-squares method, as shown in Figure 8.
(3) Fusion of PET and CT:
  After the Sym8 fusion test, practitioners believe that when a2 = 40% and b2 = 60%, the fusion image shows the absorption characteristics of PET and good CT characteristics of tissues and organs. To make it easier to distinguish, we used 256 pseudo colors for PET and fusion images, as shown in Figure 9.
(4) Lesion segmentation and labeling:
  After practitioners click anywhere on the lesion area in the PET/CT fusion image, the system grows through the PET area, maps the lesion polygon to multi-CT, forms a rectangle, divides it, and remembers to mark the corresponding description. The result is shown in Figure 10.

### 3.4. Analysis of Results

**Figure 11:**
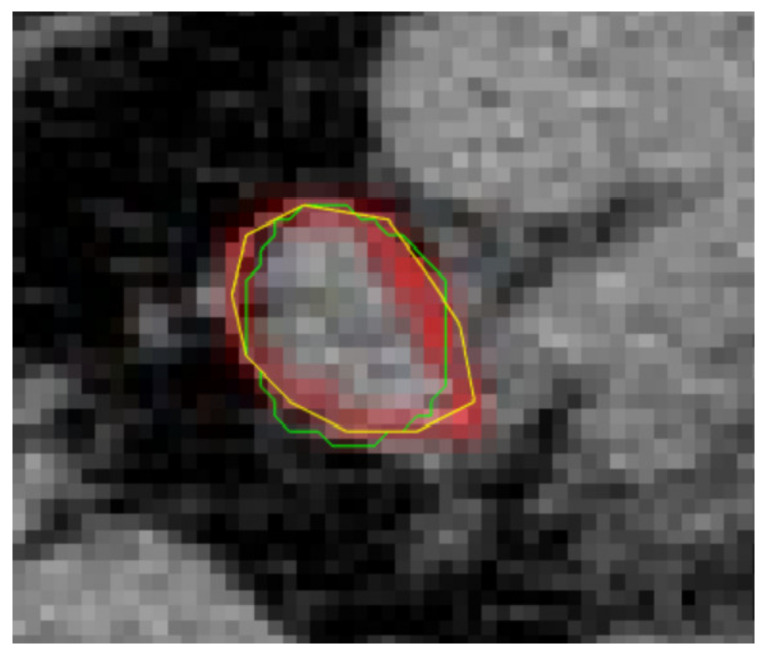
Comparison between the labeling by the proposed method and the doctor. The doctor annotated the image with a yellow line; the green line was drawn using the proposed method. After calculation with the pixels, according to the doctor’s labeling, the accuracy rate of the proposed method was 95.03%, and the error rate was 5.52%.

This study used Siemens 52-ring biograph 64 PET/CT scans from anonymous lymphoma patients. We asked clinicians with imaging expertise to label more than 100 lymphoma lesions by hand and by using our system. We randomly selected 15 CT images and compared them to the physician’s full manual labeling to verify the results (Figure 11). The system recognition rate and judgment error rate follow (Table 1):

## 4. Discussion

With the rapid development of artificial intelligence and its wide application in the medical field, identifying tumor lesions through deep learning to help doctors diagnose and treat has become an important development direction. Deep learning requires a large number of precisely labeled samples. Training samples are traditionally marked by manually delineating lesions by professional imaging doctors, and there are problems such as heavy workload and difficulty ensuring accuracy.

According to the strong absorption area of the PET and the characteristics of the CT image, the professionals randomly select a point in the focus area in the fusion image, and the system will automatically select the seed point of the focus area to delineate the tumor focus with the regional growth method. This method not only solves the problem of heavy workload but also solves the problem of automatically identifying lesions that can be misjudged. Different tissues and organs absorb imaging agents such as ^18^F-FDG in PET images. For example, the same area of high absorption (area of low gray value) is normal in some organs, while the display is abnormal in others. For example, bone and liver are strongly absorbed in this study, forming low gray areas, except for lymphatic lesions, but these are normal tissues and organs. Therefore, this method adopts a semiautomatic method; that is, a professional must roughly determine whether an area of solid absorption is a lesion, which can avoid mistaking other tissues and organs with strong but normal absorption as a lesion.

This research has some innovations in the algorithm and its implementation. In the PET linear pixel interpolation amplification and segmented grayscale transformation enhancement, the PET image reaches a size of 512 × 512 pixels, and the details of the low grayscale area are more abundant, i.e., the substantial absorption area of the imaging agent is strengthened, which is helpful for doctors to distinguish lesions. Based on gantry registration, the least-squares residual affine algorithm is used to perform more accurate registration of PET/CT images to eliminate image errors caused by long machine scanning time, human respiration, movement, etc. When Sym8 fuses PET/CT images through the ratio of 4:6, the rich details of tissues and organs in the CT images are preserved. The features of the substantial absorption area of the imaging agent can be highlighted. According to the features of the image provided by the system and through knowledge and experience, professionals can randomly select a point in the tumor focus area and the system automatically segments and labels the focus. The labeling algorithm is simple and convenient, ensuring labeling accuracy and reducing labeling workload.

This study semiautomatically performs intelligent labeling of tumor lesion samples but still needs to rely on the doctor’s professional knowledge and experience; automatic segmentation and labeling of tumor lesions on PET/CT images still needs to be tried. The critical question is how to exclude nonlesional areas with strong uptake of imaging agents.

## 5. Conclusions

This study adopted a semiautomated innovative labeling method to make CT labeling easier and more accurate on PET/CT images and to facilitate the production of deep-learning training samples. The main conclusions of this study are the following: (1) the size and signal strength of the PET image and CT image are inconsistent. To realize the registration of two images, the PET image needs to be enlarged and enhanced. This study’s linear interpolation processing algorithm is simple and has a good effect. (2) Even if the integrated PET/CT machine is used to scan the patient, the usual procedure is to scan the patient with CT first, followed by PET scanning. During the scanning, there will be a small movement because of breathing or heart beating, which results in a slight difference in the image position between the images; therefore, it is necessary to select feature points for further accurate registration in addition to the calibration of the rack. The algorithm of least-squares residual affine used in this paper performs well. (3) The characteristics of the substantial area (low gray value) of the contrast agent retained in PET and the high resolution of CT images make it easy to identify the characteristics of tissues and organs. After wavelet transformation, the effect melting of 40% and 60% is better. (4) In this study, a professional can randomly click a point in the lesion area on the PET image. The system automatically selects the seed point through the regional extreme value method, automatically delineates the focus area through the regional growth method, and maps the region to the CT image for segmentation and labeling. This method is more accurate than the automatic labeling method and easier than the completely manual labeling method.

Future research will further analyze the absorption characteristics of PET lesions to implement automatic lesion identification, improve the recognition rate, and reduce the misjudgment rate.

## Figures and Tables

**Table 1:** Comparison of the labeling results of this system and the manual labeling of professionals.

	Number of CT Images Annotated	Number of Lesions Marked	Polygonal Shape of Lesion Noted	Average Accurate Rate of Recognition (%)	Average False Positive Rate (%)
Manual labeling	15	22	various forms	100%	0%
Labeling by this system	15	22	consistent with manual labeling	93.35	6.52

Where the recognition rate = pixels correctly recognized by the system/pixels marked by hand × 100%, false positive rate = pixels misidentified by the system/pixels marked by hand × 100%.

## Data Availability

Not applicable.
